# Ten tips on how to assess bone health in patients with chronic kidney disease

**DOI:** 10.1093/ckj/sfae093

**Published:** 2024-04-15

**Authors:** Hanne Skou Jørgensen, Maria Jesús Lloret, Alexander D Lalayiannis, Rukshana Shroff, Pieter Evenepoel, European Renal Osteodystrophy (EUROD) initiative of the CKD-MBD working group of the European Renal Association (ERA), and the CKD-MBD and Dialysis working groups of the European Society of Pediatric Nephrology, Justine Bacchetta, Nathalie Bravenboer, Anibal Ferreira, Maria Fusaro, Mathias Haarhaus, Marie-Helene Lafage-Proust

**Affiliations:** Department of Clinical Medicine, Aarhus University, Aarhus, Denmark; Department of Nephrology, Aalborg University Hospital, Aalborg, Denmark; Department of Microbiology, Immunology and Transplantation, Nephrology and Renal Transplantation Research Group, Katholieke Universiteit Leuven, Leuven, Belgium; Department of Nephrology, Hospital Fundació Puigvert, Barcelona, Spain; Institut de Recerca Sant-Pau (IR-Sant Pau), Barcelona, Spain; Department of Pediatric Nephrology, Birmingham Women's and Children's Hospitals, Birmingham, UK; Renal Unit, UCL Great Ormond Street Hospital and Institute of Child Health, London, UK; Department of Microbiology, Immunology and Transplantation, Nephrology and Renal Transplantation Research Group, Katholieke Universiteit Leuven, Leuven, Belgium; Department of Nephrology and Renal Transplantation, University Hospitals Leuven, Leuven, Belgium

**Keywords:** bone density, chronic kidney disease, mineral and bone disorder, fracture, osteoporosis, renal osteodystrophy

## Abstract

Patients with chronic kidney disease (CKD) experience a several-fold increased risk of fracture. Despite the high incidence and the associated excess morbidity and premature mortality, bone fragility in CKD, or CKD-associated osteoporosis, remains a blind spot in nephrology with an immense treatment gap. Defining the bone phenotype is a prerequisite for the appropriate therapy of CKD-associated osteoporosis at the patient level. In the present review, we suggest 10 practical ‘tips and tricks’ for the assessment of bone health in patients with CKD. We describe the clinical, biochemical, and radiological evaluation of bone health, alongside the benefits and limitations of the available diagnostics. A bone biopsy, the gold standard for diagnosing renal bone disease, is invasive and not widely available; although useful in complex cases, we do not consider it an essential component of bone assessment in patients with CKD-associated osteoporosis. Furthermore, we advocate for the deployment of multidisciplinary expert teams at local, national, and potentially international level. Finally, we address the knowledge gaps in the diagnosis, particularly early detection, appropriate “real-time” monitoring of bone health in this highly vulnerable population, and emerging diagnostic tools, currently primarily used in research, that may be on the horizon of clinical practice.

## INTRODUCTION

In patients with chronic kidney disease (CKD), optimal control of mineral and bone disorder (MBD) is important not only to prevent debilitating skeletal complications, but also for preserving cardiovascular health [1]. Despite an overall improvement in CKD-MBD care, patients with CKD still experience a multifold increased fracture risk compared to age and sex matched controls [2–4]. The risk of mortality following a hip fracture remains substantially higher in people with CKD [5]. The risk of rehospitalization, is almost 4-fold higher [5]. The prevalence of vertebral fractures is similarly high at 18% in patients with CKD G3-5, and has been identified as an independent predictor of all-cause mortality [6].

Despite the high risk and poor outcomes, bone fragility in CKD, also referred to as CKD-associated osteoporosis, is poorly studied, with a paucity of systematic reviews, meta-analyses, and randomized controlled trials addressing diagnostics or treatment. So far, patients with CKD have been excluded from all registration trials evaluating novel pharmacotherapies.

We describe the clinical, biochemical, and radiological evaluation of bone health, with the benefits and limitations of these, as well as newer research tools. Bone histomorphometry, although the gold standard for diagnosing renal bone disease, is invasive, cannot be used for rapid decision-making, requires specialist histological expertise, and is available only at specialist centers worldwide. It is not easily implemented as a disease and treatment monitoring tool, and we do not include this as an essential component of bone assessment. Evaluation of bone health in children with CKD is briefly considered here, with further details described in European guidelines [7–10]. This document focuses on CKD-MBD assessment in adults.

### Be aware of the fracture burden in patients with CKD

It is important to be aware of the immense fracture burden and related morbidity and mortality in patients with CKD. As CKD progresses, both bone quantity and quality are impaired, enhancing the risk of fractures. Fracture incidence increases steadily as kidney function declines from 15 to 20, 24, 31, and 46 per 1000 person-years for CKD G1-2, 3A, 3B, and 4, respectively [11]. This is particularly true for CKD patients older than 65 years, with 10% of women and 5% of men experiencing at least one fracture during 3 years of follow-up [12]. The risk of skeletal fracture is at least 4 times higher in patients with CKDG5-5D compared to individuals with normal kidney function [2, 3]. The most common sites of fracture are the femur (neck or intertrochanteric region), forearm, and humerus. Vertebral fractures are also very common in patients with CKD, but frequently underdiagnosed as many are asymptomatic [13]. Hip fractures are especially concerning as they are related to long hospital stays and high subsequent mortality [5]. Falls are an important risk factor for fractures, [14] and patients with CKD are more prone to falls than patients without CKD [15]. Falls result from a complex interaction of multiple factors including general frailty, muscle weakness, impaired mobility, cognitive decline, neuropathy, and polypharmacy, all of which are highly prevalent in patients with CKD [16]. The fear of falling can lead to decreased physical activity, further increasing the risk. Risk screening should be considered for patients older than 50 years, for postmenopausal women, and in specific high-risk situations (systemic glucocorticoid therapy, musculoskeletal symptoms) [17]. Risk assessment should include history of fracture, individual risk factors, and bone imaging, as discussed next.

**Figure:**
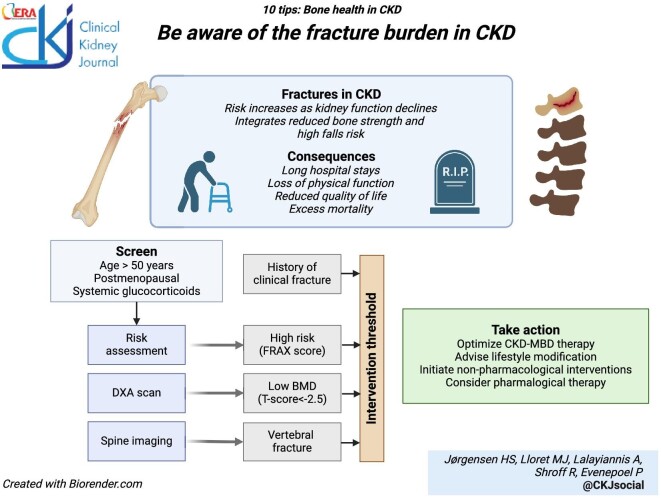


### Define the risk profile with the aid of a fracture prediction tool

Both traditional and non-traditional risk factors contribute to bone fragility in CKD, [18, 19] and these need to be assessed at an individual level. Older age, female sex, and Caucasian ethnicity all increase the risk of fractures in CKD, [5, 14] as do lifestyle factors such as smoking [20]. CKD-associated risk factors include MBD, acidosis, chronic inflammation, and age at presentation and duration of disease. Poor growth remains one of the most widely prevalent manifestations of childhood CKD with 50% of children failing to reach their full height potential, [21] and short stature increasing the risk of hospitalization, morbidity, and mortality [22]. Bone pain is frequently reported in children with CKD, [23] and limb deformities, fractures and radiological signs of bone disease are noted in ∼15% of children on peritoneal dialysis [24]. In adults with childhood-onset CKD, peak bone mass may be adversely affected, resulting in increased bone fragility in adulthood [25, 26]. The underlying etiology of CKD may also influence the risk of MBD. Patients with autosomal dominant polycystic kidney disease exhibit a particular bone phenotype characterized by low bone turnover with preserved bone mass [27, 28]. Diabetes mellitus adversely affects bone quality, with increased fracture risk both for type 1 and type 2 diabetes [29, 30]. Other systemic diseases such as rheumatological [31, 32], hematological, gastrointestinal, and endocrine disorders [33] may all affect the skeleton (Table 1).

**Figure:**
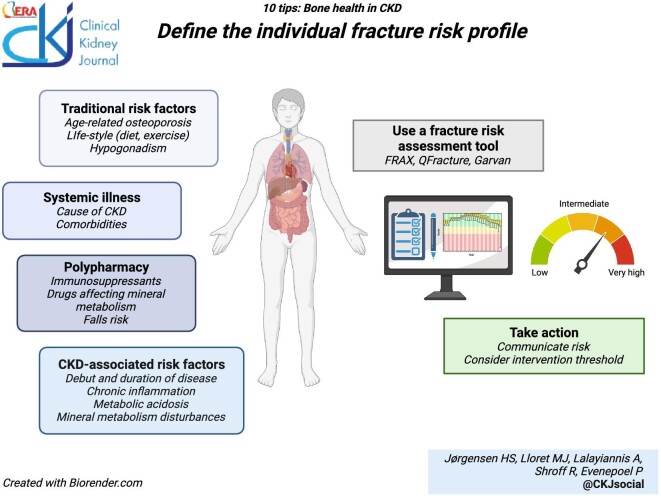


**Table 1:** Effect of systemic disorders on bone health.

Organ system	Disease	Effect on bone health
Endocrine	Diabetes mellitus type 1 and 2	Low bone turnover and reduced bone quality; low bone density in type 1 [30,31]
	Cortisol excess (Cushing's disease)	Low bone turnover bone loss; impaired calcium absorption [149]
	Growth hormone excess	High bone turnover; defects mainly in trabecular bone [150]
	Hyperparathyroidism	High bone turnover; defects mainly in cortical bone [151, 152]
	Hyperthyroidism	Unbalanced bone remodeling with high bone resorption; hypercalciuria with negative calcium balance [153]
	Hypogonadism	Unbalanced bone remodeling with high bone resorption [154, 155]
	Primary hyperaldosteronism	Hypercalciuria with negative calcium balance [156]
Rheumatological	Ankylosing spondylitisConnective tissue diseasesRheumatoid arthritisSystemic lupus erythematosus	Multifactorial background (genetic predisposition, serological factors, hormonal disturbances); systemic inflammation likely plays a key role [157, 158]
Hematological	Monoclonal gammopathies	Osteolytic lesions; circulating cytokines may exert additional negative effects [159, 160]
Gastrointestinal	Chronic liver diseaseCoeliac diseaseInflammatory bowel diseasePrimary biliary cholangitis	Malnutrition with insufficient intake of calcium and vitamin D; systemic inflammation may play a role [161, 162]

Fracture risk prediction tools such as FRAX^®^, QFracture^®^, and Garvan are convenient to assess the contributions of traditional risk factors for fractures in adults [34]. Although CKD is not included as a cause of secondary osteoporosis in these tools, FRAX^®^ (https://www.sheffield.ac.uk/FRAX) has been shown to perform acceptably for patients with CKD [35–38]. The tool is easily accessed through the website or as a desktop application and can be used as an aid to communicating risk, as it delivers 10-year probabilities of a major osteoporotic fracture or a hip fracture. Although risk of falling contributes greatly to fracture risk [39, 40, 41], falls are not currently included in the FRAX^®^ tool. However, the recently launched FRAX^®^plus (https://fraxplus.org/) includes modification of the risk estimates by number of falls in the previous year [42]. The overall physical function of the patient, discussed next, is highly relevant for falls risk. Last, a previous fracture, particularly if caused by low-energy trauma implies bone fragility and is a strong predictor of future fractures, [12, 43, 44] and symptoms such as bone pain or spontaneous back pain should prompt work-up for bone mineralization defects or spontaneous vertebral fractures.

### Review medications and limit exposure to drugs affecting bone health

Patients with CKD are frequently exposed to polypharmacy, including drugs that are known to affect bone metabolism. Systemic therapy with *glucocorticoids*, commonly used in combination with other immunosuppressants for glomerular disorders [45] and after kidney transplantation, [46, 47] has well-known negative effects on bone health. Drugs affecting the central nervous system increase the risk of falling, thereby contributing to fracture risk [48]. Common drugs such as diuretics, proton pump inhibitors and anti-coagulants may affect MBD, though the effects on fracture risk in CKD is less certain (Table 2). While many of these drugs have valid indications in CKD, it is important to be aware of their “osteotoxic” risk and take steps to reduce any negative effects on bone health. Exposure should be limited whenever feasible, by considering treatment duration, dosages, and indications for discontinuation. For potent drugs such as systemic glucocorticoids, fracture risk should be assessed when initiating therapy, with guidelines recommending non-pharmacological interventions and calcium and vitamin D supplementation for all patients [49, 50], and anti-resorptive therapy for those at very high risk [51].

**Figure:**
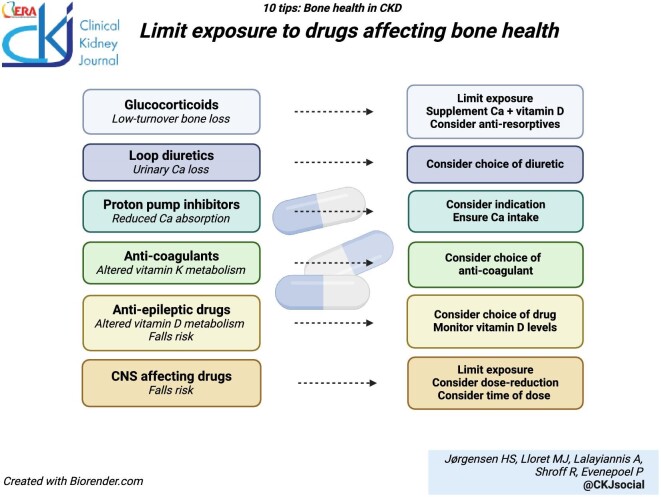


**Table 2:** Medications affecting mineral metabolism, bone health or fracture risk.

Class of drugs	Mechanism	Action
Systemic glucocorticoids	Low turnover bone loss [163, 164]; inhibits bone formation and stimulates bone resorption	Limit exposure (duration and dose): consider steroid-sparing (combination) immunosuppressive regimens
	Fracture risk increased with both higher doses and longer exposure [165, 166]	Supplement calcium and vitamin DConsider anti-resorptive therapy
Medications affecting the central nervous system (anti-depressants, benzodiazepines, narcotics)	Risk of falling; increased fracture risk in the background population [48]	Limit exposureConsider dose-reduction in CKDConsider time of dose (bedtime)
Anti-epileptic drugs	Vitamin D metabolism; reduced levels of vitamin D [167, 168]	Consider choice of anti-epileptic. Monitor and supplement vitamin D levels
Diuretics	Calcium metabolism; increased urinary calcium loss with loop-diuretics; calcium retention with thiazides	Consider choice of diuretic
	Fracture risk increased with loop-diuretics [2, 48] and decreased with thiazides [169, 170] in the general population	Consider total calcium intake
Proton pump inhibitors	Reduced absorption of cations such as calcium and magnesium; possibly direct bone toxicity. Increased fracture risk both in the general population [48] and in CKD [171, 172]	Limit exposure (short courses, plan for discontinuation)Consider total calcium intake
Anti-coagulants (Vitamin K antagonists)	Vitamin K metabolism; reduced action of vitamin K dependent proteins [173, 174] Effect on bone health unclear [175, 176].	Consider choice of anticoagulant

### Assess musculoskeletal function and frailty

Mechanical loading is key for the development and maintenance of bone strength throughout life [52, 53]. Conversely, loss of muscle mass and function, sarcopenia, contributes to falls, fractures, and overall poor outcomes both in the general population [54] and in CKD [40]. Of interest, body composition can be measured by dual-energy X-ray absorptiometry (DXA) or computed tomography (CT) enabling simultaneous evaluation of bone and muscle mass [55]. Both self-reported and measured physical function associate with risk of falls and fractures in CKD [56, 57]. Physical performance batteries can be used to assess the function of the musculoskeletal system through a set of short exercises (gait speed, balance tests, standing up from a chair) [58, 59]. Poor physical function can also be addressed—positive musculoskeletal effects of exercise are seen even in patients receiving maintenance dialysis [60–63]. A broader assessment of general frailty, [64, 65] for example by the Fried Frailty Phenotype (weight loss, fatigue, decreased grip strength, slow gait speed, low physical activity) [66] may also be useful for the risk/benefit assessment and shared decision-making of medical and non-medical interventions.

**Figure:**
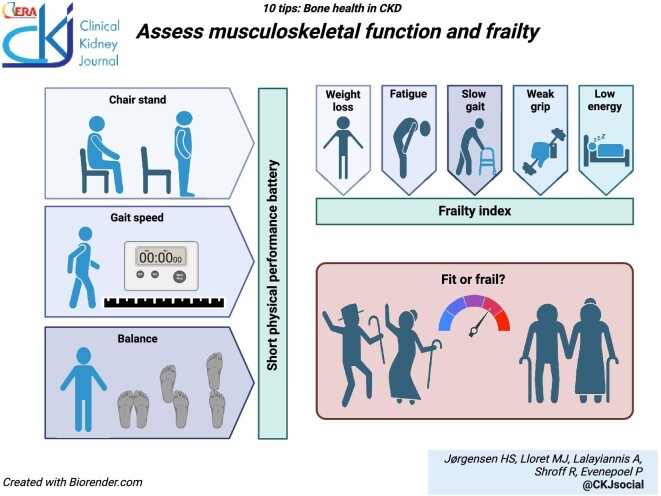


### Provide sufficient dietary calcium and vitamin D

Epidemiological studies estimate that the average dietary calcium intake in adults with CKD G5–5D is between 400–800 mg/day, [67] which is well below recommended levels for the overall population [68]. In CKD, dysregulated calcium homeostasis increases the risk of either a negative or a positive calcium balance. Overt calcium deficiency potentially contributes to inadequate control of hyperparathyroidism, increasing fracture risk, whereas excess calcium can lead to vascular calcification [69] and abnormal cardiac structure and function [70]. The dietary calcium intake, [71, 72] as well as its bioavailability, [73–75] typically decreases with age and the progression of CKD; the fractional intestinal absorption of calcium is approximately 15% in the CKD population. For rapid estimation of calcium intake, self-administered online calculators from the International Osteoporosis Foundation [https://www.osteoporosis.foundation/educational-hub/topic/calcium-calculator], the Royal Osteoporosis Society [https://webapps.igc.ed.ac.uk/world/research/rheumatological/calcium-calculator] [76, 77], or national societies that include local dietary preferences are quick and easy to use.

Calcium-containing phosphate binders are an important source of calcium in patients with CKD G4-5D and can contribute up to 70% of the overall calcium intake [71, 78–80]. However, with a growing awareness of vascular calcification, there has been a move away from calcium-based therapies, rendering patients at risk of a negative calcium balance. Accordingly, a recent European consensus statement has suggested a minimum total calcium intake from diet and medications of 800–1000 mg/d, and not exceeding 1500 mg/d, to maintain a neutral calcium balance in adults with CKD [67]. These recommendations reflect the calcium requirements for healthy adults over 25 years of age, as well as the recommended calcium intake suggested by the KDOQI clinical practice guidelines on nutrition [81]. In children with CKD, total calcium intake should be kept within the age-appropriate normal range [67, 82]. In some circumstances, such as in physiological conditions requiring additional calcium supply (pregnancy and lactation), intensified dialysis regimens, or ‘hungry bone syndrome’, a higher calcium intake may be required.

In adults with CKD, active vitamin D compounds have failed to improve outcomes beyond PTH control. Hence, current guidelines state that it is reasonable to reserve the use of calcitriol and active vitamin D compounds for patients with advanced CKD and severe and progressive hyperparathyroidism [83] although more targeted PTH control during non-dialysis-CKD period may influence outcomes during HD [84]. Nutritional vitamin D supplementation is recommended as in the general population. In children with CKD, European consensus guidelines recommend supplementation with nutritional vitamin D to maintain serum 25-hydroxyvitamin D levels >75 nmol/L (>30 ng/ml) and use of active vitamin D analogs to prevent hypocalcemia and hyperparathyroidism [8].

### Monitor biomarkers of mineral and bone disorders

The Kidney Disease Improving Global Outcomes (KDIGO) 2017 guideline on the management of CKD-MBD recommend that in patients with CKD G3a–G5D, treatment of CKD-MBD should be based on serial assessments of calcium, phosphate, and PTH levels, considered together [85]. These parameters, complemented, by 25-hydroxyvitamin D, alkaline phosphatase (ALP) and serum bicarbonate levels, form the mainstay of MBD assessment in routine clinical practice. However, poor sensitivity and specificity, as well as analytical heterogeneity and biological variability, limit their capability to identify the bone phenotype at an individual level. Moreover, in patients with long-standing hyperparathyroidism, PTH hyporesponsiveness, or a desensitization of the skeleton, may develop, reducing the diagnostic accuracy of PTH levels in the evaluation of bone turnover [86].

During the process of skeletal remodeling, biomarkers of bone formation (bone ALP, trimeric procollagen type I N-terminal propeptide) and resorption (tartrate-resistant acid phosphatase isoform 5b) are released from the bone, and circulating levels can be used as a non-invasive measure of overall skeletal turnover [87]. These biomarkers passively reflect the ‘real-time’ state of bone turnover independent of any underlying causes such as PTH levels [86–88]. Thus, rather than relying on PTH levels alone, a combination of PTH and bone turnover markers has been shown to improve diagnostic accuracy of low vs non-low and high vs non-high bone turnover [86]. The diagnostic utility of biomarkers in distinguishing high and low turnover bone disease has been documented compared with bone histomorphometry and high-resolution peripheral quantitative CT (HR-pQCT) [86, 89, 90]. Bone-specific ALP represents ∼50% of total circulating ALP so that, except in patients with severe liver disease, total ALP may be considered an acceptable marker of bone formation and mineralization [91]. Metabolic acidosis can induce bone demineralization and the release of calcium from the skeleton by a direct physiochemical effect as well as by stimulating osteoclast-mediated bone resorption and inhibiting osteoblastic differentiation [92]. Therefore, serum bicarbonate levels should be regularly monitored and repletion considered.

### Assess bone mass by dual-energy X-ray absorptiometry

DXA is widely available and the clinical standard to measure bone mineral density (BMD) as a proxy of bone mass or quantity. DXA predicts fractures in CKD [93–95] as well as in the non-CKD population. DXA testing should be considered in patients with CKD aged >50 years and in women who are postmenopausal [96]. It is important to acknowledge that DXA does not have sufficient resolution to discriminate between cortical and trabecular bone or between deficits in bone volume and mineralization. As in the general population, the hip and the lumbar spine are the primary skeletal sites to evaluate. In cases where spine imaging is not assessable, the ultradistal radius may be useful to evaluate trabecular bone, and BMD at the distal one-third radius may be particularly interesting in CKD, as it informs on cortical bone. For the lumbar spine, well-known sources of bias include deformities in the form of vertebral fractures, scoliosis, or degenerative and sclerotic bone disease [97]. Specifically to CKD, peritoneal dialysate [98] and mineral-containing phosphate binders within the gastrointestinal tract [99] may interfere with imaging. Theoretically, so can aortic calcification, although the effect is small and unlikely to be of clinical relevance [100, 101]. A functioning arteriovenous fistula may affect radial BMD measurements, so measurements on the contralateral arm are preferred [102, 103]. DXA should only be repeated if the result will influence clinical management or if a change in BMD exceeding the least significant change (∼2–3%) is expected [104].

A low threshold should be adopted for screening for vertebral fractures in patients with CKD, as these are common, often remain undiagnosed, and signals a high risk of future fractures [6, 13, 105]. Vertebral fracture assessment either by lateral X-ray or DXA of the spine also allows assessment of abdominal aortic calcification [106] and thus may be useful in concomitantly stratifying cardiovascular risk [107, 108]. Screening for vertebral fractures can be recommended in the following situations: patients with height loss >4 cm or marked kyphosis, a history of vertebral fracture or recent back pain, and current or previous glucocorticoid exposure.

There is no evidence to recommend routine DXA in children with CKD. Given that DXA measures areal BMD, it can underestimate the true volumetric BMD in children with short stature or overestimate BMD in a tall child, making serial DXA scanning in growing children particularly challenging [23, 109].

### Consider novel imaging techniques and analyses of bone strength

Bone strength is the capacity to resist trauma without fracture. Both bone quantity, as measured by BMD, and bone quality contributes to overall bone strength [110]. Bone quality is determined by bone geometry, microarchitecture, and composition. Each of these characteristics contain subparameters such as hip axis length and shaft angle, cortical and trabecular thickness, porosity and connectivity, and matrix composition, collagen fiber arrangement, and mineralization. Derived from these parameters are estimates of bone strength and load-bearing capacity [111, 112].

Quantitative CT (QCT), peripheral QCT (pQCT), and HR-pQCT are three-dimensional imaging techniques that deliver information on volumetric BMD of cortical and trabecular bone separately, at the axial (QCT) or distal (pQCT, HR-pQCT) skeleton. HR-pQCT additionally permits detailed analyses of bone microarchitecture, including trabecular connectivity and cortical thickness and porosity. CKD associates with bone micro-architectural impairment even at normal BMD, [113] and rapid cortical bone loss [114]; however, cortical porosity has not been shown to be superior in discriminating fracture status in CKD [115].

Complementary techniques that estimate bone strength by conventional bone imaging have been developed including trabecular bone score (TBS), hip structural analysis (HAS), and 3D-DXA from DXA images and finite element (FE) analysis from high-resolution imaging modalities. TBS performs gray-level texture measurements on lumbar spine images, capturing information relating to trabecular microarchitecture. In the general population, TBS adds information on fracture risk independently of BMD and other risk factors (e.g. FRAX^®^ score) [116]. TBS also discriminates fracture status in adults and children receiving dialysis [117, 118] and may prove to be a useful adjunct to BMD for fracture prediction in CKD [119–121]. HSA allows for the description of geometric characteristics of the proximal femur, which may have implications for bone strength, potentially improving fracture risk prediction at this important skeletal site [122, 123]. Last, 3D-DXA modeling aims to deliver similar information as QCT at lower radiation exposure, [124] by providing estimates of trabecular and cortical volumetric BMD and cortical thickness at the hip through computational analysis of the standard DXA images [125].

FE analysis can be used to compute bone mechanical properties from QCT, HR-pQCT, or magnetic resonance scans. Bone structures are identified from the images and transformed into voxel-based FE models with assigned material properties [126]. Load simulations can be performed to estimate conditions at which structural failure will occur. Smaller studies validating these measures against other imaging techniques or bone histomorphometry in CKD have been published, [127, 128] but data on clinical utility are not yet available.

Radiofrequency echographic multi-spectrometry (REMS) is an ultrasound technology applied to axial sites (hip and spine) that delivers both BMD and bone strength estimation [112]. The ultrasound signals backscattered from the bone structures are analyzed and the pattern compared with models of fractured and non-fractured patients. The resulting fragility score relates to bone microarchitecture and may improve fracture prediction [129].

These newer techniques and options of image analysis show promise as they may provide more in-depth information on bone health with little additional cost. However, further validation will be necessary before implementation in clinical practice.

### Consider a bone biopsy for complex cases

A bone biopsy remains the gold standard to assess renal bone disease, providing information not only on bone turnover, but also on bone mineralization and cortical and trabecular microarchitecture [130]. Bone biopsies are obtained at the iliac crest, except for surgical samples taken at other sites. Given the heterogeneity of bone sites (weight bearing vs non-weight bearing, cortical vs trabecular predominance), some caution is warranted when extrapolating results to the overall status of the skeleton. Taking a bone biopsy is time-consuming, and the subsequent analysis of the sample is expensive and necessitates specific histopathological expertise, which is not widely available [131]. As a result, the complexity of the procedure often results in diagnostic delay, which is a limiting factor for patients at imminent risk of fracture who require a prompt diagnosis. The procedure is invasive, and further important limitations include associated pain and risk of complications. Performing the puncture with a smaller internal diameter needle (<5 mm rather than 8 mm) may reduce these risks and seems adequate for diagnostic purposes [132]. In recent studies, the complication rate following diagnostic bone biopsies with such smaller needles have been reported to be ∼3–4% [86]. It is important to emphasize that the KDIGO guidelines on CKD-MBD management no longer recommend that a bone biopsy should be performed before initiating bone-targeting therapies in CKD [85]. Therefore, a bone biopsy should not be considered a mandatory step in the evaluation of bone health. However, it may be reasonable to perform a bone biopsy in complex cases: to exclude a bone mineralization defect, to confirm suspicions of low bone turnover, or to rule out atypical bone pathology, as these are likely to require a different therapeutic approach [133]. Examples of patients who fall into this category are those with symptoms or biochemistry indicative of a mineralization defect (bone pain, multiple fractures, atypical fractures, chronic hypocalcemia, hypophosphatemia, or hypovitaminosis D); those with *a priori* suspicion of low bone turnover (previous parathyroidectomy, low bone turnover markers, or discrepancies between MBD and bone turnover markers), and those with important comorbidities that could indicate unusual bone pathology (see next paragraph).

### Seek help: the multidisciplinary team approach

The tips included so far (Table 3) should enable a comprehensive phenotyping of bone health and provide therapeutic guidance for most patients with CKD presenting with bone fragility. However, considering the diverse manifestations of MBD in CKD, as well as the multi-morbidity of our patients, complex and challenging cases may benefit from a multidisciplinary team (MDT) approach [134]. Patients who may be considered include those with a history of atypical or multiple fractures, therapy failure, divergent biomarker findings, atypical mineral metabolism profiles, or important comorbidities such as malnutrition, endocrine disturbances, etc. Expert MDTs may be deployed at the local, regional, national, or even international level and should include nephrologists with expertise in CKD-MBD and bone experts from other fields, such as rheumatologists, endocrinologists, or geriatricians. Additional advice may be sought from pathologists, radiologists, and endocrine and/or orthopedic surgeons. Ideally, the MDT should have access to bone histomorphometry and other expert diagnostic options. An expert MDT is complimentary to the Fracture Liaison Service, a multidisciplinary and multiprofessional team already operational in many hospitals, focusing on secondary preventive care following a fracture, often bridging surgical and medical specialties [135].

**Figure:**
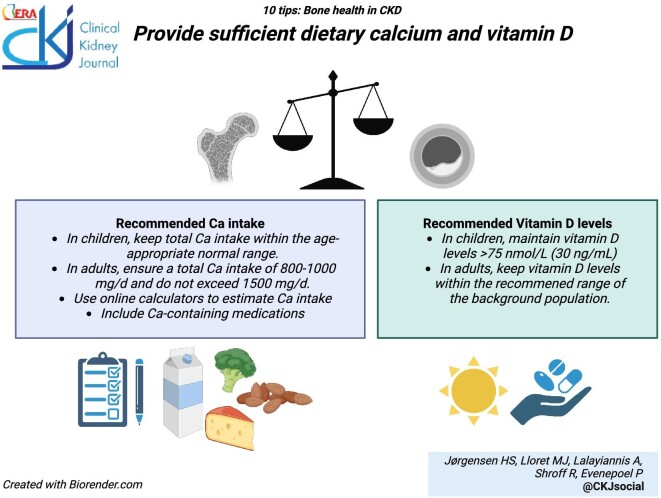


**Figure:**
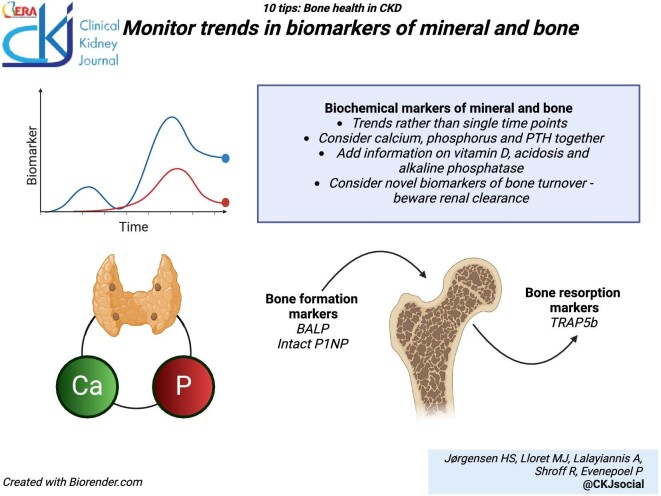


**Figure:**
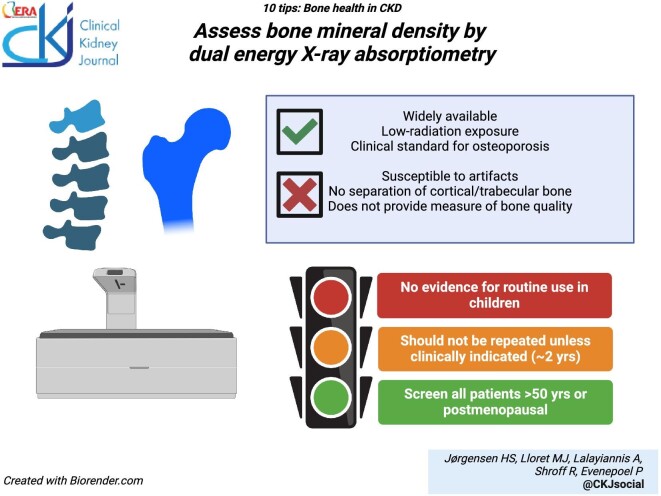


**Figure:**
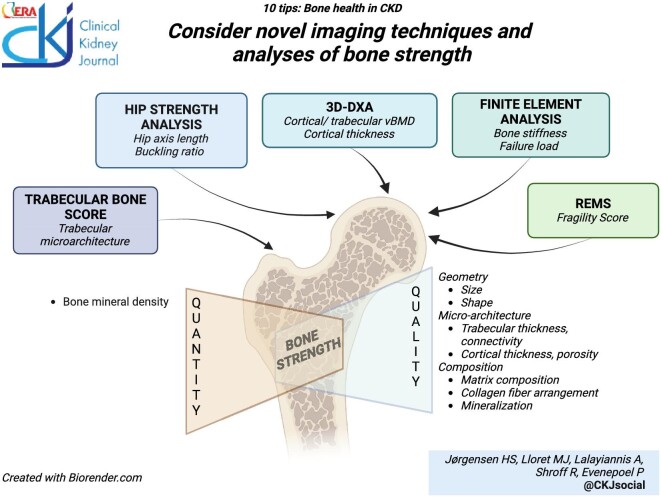


**Figure:**
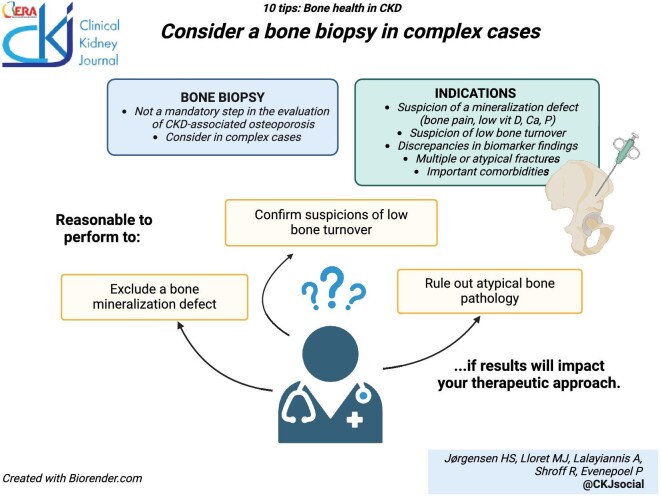


**Figure:**
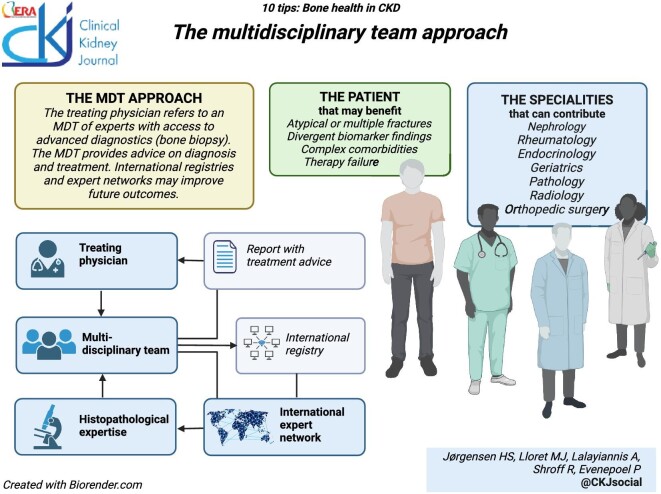


**Table 3:** “Do” and “Do not” tips when assessing bone health in CKD.

	Do	Do not
Clinical History	Be aware of the fracture burden in patients with CKD	Do not neglect fracture history
Consider fracture risk	Assess risk factors: traditional and CKD-associatedUse FRAX^®^ or other fracture prediction tools	Do not ignore musculoskeletal symptoms and silent vertebral fractures
Medications	Consider corticosteroid-sparing strategiesConsider indication, dosage and timing of agents that may increase falls riskConsider discontinuing other agents that may negatively affect bone health	Do not prescribe systemic glucocorticoids without fracture risk assessment
Musculoskeletal function	Assess physical performance and overall frailtyEncourage increased exercise	Do not neglect the importance of recent falls
Dietary Calcium and Vitamin D intake	Assess dietary Ca intakeMonitor 25OHD	Do not monitor 1,25(OH)_2_D
Biomarkers of MBD	Monitor PTH, ALP, Ca, and P routinely	Do not measure sex steroids
	Include 25OHD and serum bicarbonateConsider biomarkers of bone formation (BALP, intact PINP) and resorption (TRAP5b) to guide therapy and monitor treatment response	Do not neglect that traditional bone turnover markers are cleared by the kidneys (CTX, total PINP)
Imaging	Consider routine DXA for patients >50 years or postmenopausal	Do not perform DXA without a plan of action for the results
	Include vertebral fracture assessment Consider lateral X-ray for prevalent vertebral fractures and abdominal aortic calcification	
Novel imaging (QCT, pQCT, HR-pQCT)	Consider adding information from bone strength analyses available from traditional bone imaging	Do not use routinely
	Consider pQCT if available for information on cortical bone	
Bone histomorphometry	Consider in complex cases	Do not use routinely
Multidisciplinary team	Use the expertise available in an MDT (dietician, rheumatology, endocrinology, surgery, and others)	Do not succumb to nihilism

*Abbreviations:* Ca = calcium, P = phosphate, ALP = alkaline phosphatase, BALP = bone-specific alkaline phosphatase, CTX = C-terminal telopeptide of type I collagen, PINP = procollagen type I N-terminal propeptide, PTH = parathyroid hormone, pQCT = peripheral quantitative computed tomography, QCT = quantitative computed tomography, TRAP5b = tartrate-resistant acid phosphatase isoform 5b

## RESEARCH RECOMMENDATIONS

1. **Calcium isotope diagnostics.** The ratio of naturally-occurring, non-radioactive isotopes of calcium has been shown to be a sensitive biomarker of bone calcium balance (BCaB). In children with CKD or on dialysis, serum Ca isotope ratio was a significant and independent predictor of BCaB, correlating with bone densitometry and biomarkers of bone formation and resorption. Children with CKD and on dialysis had a significantly lower Ca isotope ratio compared to their healthy peers, reflecting low BCaB [136], and isotope ratios were even lower than values reported in elderly osteoporotic women [137]. Ca isotope diagnostics are currently a research tool, but as part of a comprehensive bone biomarker panel they may prove to be a helpful adjunct to current diagnostics, enabling the early identification of patients who may benefit from osteoporosis treatment, and allowing real-time monitoring of the therapeutic response and predicting fracture risk.
2. **Bone impact microindentation** (IMI) is a novel technique based on the principle that the deeper a probe penetrates the bone surface, the lower the resistance of the bone to mechanical impact [138]. Thus, IMI might offer the possibility of assessing the mechanical properties of cortical bone, which is often affected in CKD [139]. IMI is performed with a handheld device (Osteoprobe®). Reduced bone material strength index measured by IMI is associated with both increased risk and greater severity of osteoporotic fractures, [140] independently of BMD [141]. IMI may be particularly useful in cases of secondary osteoporosis and metabolic bone disorders, including CKD [142], but there is currently insufficient evidence to support its introduction into clinical practice.
3. **^18^F-sodium fluoride positron emission tomography** (^18^F-NaF-PET) uses a bone-seeking tracer (^18^F-fluoride) to identify sites of active bone remodeling. The use of modern PET/CT systems has greatly improved the resolution of ^18^F-fluoride images and consequently, utility has expanded beyond detection of skeletal malignancies to include inflammatory lesions, avascular necrosis and metabolic bone diseases [143]. In CKD, ^18^F-fluoride activity correlates strongly with bone histomorphometric parameters of skeletal remodeling, with very good diagnostic accuracy for high and low bone turnover [144–146]. Of interest, ^18^F-NaF-PET also identifies sites of active vascular calcification, [147] and may detect early vascular lesions before arterial wall calcifications can be visualized by CT [148]. By providing simultaneous assessment of active vascular calcification and skeletal remodeling, ^18^F-NaF-PET could offer exciting research possibilities in the field of CKD-MBD.

## Data Availability

No new data were generated or analyzed in support of this research.
